# Aging and the relationships between long-axis systolic and early diastolic excursion, isovolumic relaxation time and left ventricular length—Implications for the interpretation of aging effects on e`

**DOI:** 10.1371/journal.pone.0210277

**Published:** 2019-01-07

**Authors:** Roger E. Peverill

**Affiliations:** Monash Cardiovascular Research Centre, MonashHeart and Department of Medicine (School of Clinical Sciences at Monash Health), Monash University and Monash Health, Clayton, Victoria, Australia; Medical College of Georgia at Augusta University, UNITED STATES

## Abstract

**Background:**

Both the left ventricular (LV) long-axis peak early diastolic lengthening velocity (e`) and long-axis early diastolic excursion (EDExc) decrease with age, but the mechanisms underlying these decreases are not fully understood. The aim of this study was to investigate the relative contributions to aging-related decreases in e`and EDExc from LV long-axis systolic excursion (SExc), isovolumic relaxation time (IVRT, as a measure of the speed of relaxation) and LV end-diastolic length (LVEDL).

**Methods:**

The study group was 50 healthy adult subjects of ages 17–75 years with a normal LV ejection fraction. SExc, EDExc, e`and IVRT were measured from pulsed wave tissue Doppler signals acquired from the septal and lateral walls. Multivariate modelling was performed to identify independent predictors of EDExc and e`which were consistent for the septal and lateral walls.

**Results:**

EDExc decreased with age and the major determinant of EDExc was SExc, which also decreased with age. There was also a decrease of e`with age, and the major determinant of e`was EDExc. IVRT decreased with age and on univariate analysis was not only inversely correlated with EDExc and e`, but also with SExc. IVRT was only a minor contributor to models of EDExc which included SExc, and was an inconsistent contributor to models of e`which included EDExc. LVEDL decreased with age independent of sex and body size, and was positively correlated with SExc, EDExc and e`.

**Conclusion:**

Major mechanisms underlying the decrease in e`seen during aging are the concomitant decreases in long-axis contraction and early diastolic excursion, which are in turn related in part to long-axis remodelling of the left ventricle. After adjusting for the extent of systolic and early diastolic excursion, slowing of relaxation, as reflected in prolongation of the IVRT, makes no more than a minor contribution to aging-related decreases in EDExc and e`.

## Introduction

Studies performed in adult human subjects in the 1960s, 1970s and 1980s demonstrated that delay in the onset [1, 2], a reduced maximum velocity [3–6], and a decrease in the extent of LV early diastolic filling [5–7] can occur despite the presence of a normal left ventricular (LV) ejection fraction. An important implication of these findings was that early diastolic variables provide a more sensitive means of identifying LV myocardial dysfunction than ejection fraction. A more recent example of this phenomenon is the aging-related decrease in the LV long-axis tissue Doppler imaging (TDI) peak early diastolic lengthening velocity (e`) which begins early in the adults years and continues throughout life [8–15]. The reduction in e`with aging has been attributed to a slowing of LV relaxation [12, 16], a mechanism which is also believed to explain the aging-related prolongation of the isovolumic relaxation time (IVRT) [17]. Consistent with slowing of LV relaxation being a mechanism for e`changes with aging are studies showing an inverse correlation of e`with the invasively derived time constant of relaxation (tau) [18–21]. However, the evidence from invasive studies regarding the relationship of tau with age has been inconsistent [22, 23], and e`appears to be only weakly correlated with tau in subjects with a normal ejection fraction [21]. Moreover, there is evidence that factors other than the speed of relaxation can affect e`, including that e`is correlated with the peak velocity of long-axis contraction (s`) [15, 24] and the extent of long-axis contraction [25], and that changes in e`during inotropic interventions appear to be determined in large part by changes in the extent of the preceding contraction [26, 27].

Most of the focus of research on diastolic function over the last 40 years has been on the maximum velocities of LV filling and motion, but given that a fundamental element of LV relaxation is the return of the myocardium in the ventricle to its pre-contraction length [28], it is likely that measures of maximal velocity alone do not provide a complete description of the relaxation process. It has even been suggested that the extent of relaxation may have more significance for cardiac function than the speed of relaxation [28], yet despite this, the extent of early diastolic motion has been a relatively uncommon inclusion in studies of LV relaxation [6, 24, 29–32]. There have been isolated reports that aging is not only accompanied by a reduction in e`but also by a reduction in the extent of early diastolic excursion (EDExc) [32, 33]. In addition, there is increasing evidence that aging is accompanied by a gradual decrease in LV end-diastolic length (LVEDL) [32, 34–36], and one report that aging-related decreases in LVEDL and increases in blood pressure (BP) can account for a proportion of the aging-related decreases in both EDExc and e`[33]. Given that EDExc must be intrinsically limited by the extent of LV long-axis systolic excursion (SExc), it is important that reductions in SExc with age have also been reported [30, 32, 37–40]. However, the relationships of EDExc and e`with age, LVEDL, BP and SExc, and the inter-relationships of these variables, have not been systematically examined. Neither has there been any specific investigation of how IVRT, a variable dependent on the speed of LV relaxation [41], might relate to EDExc and e`.

Accordingly, the aim of this study was to investigate the relationships of SExc, BP, LVEDL and IVRT with EDExc and e`in a healthy cohort of subjects covering the spectrum of ages from young adult to elderly. The study group was 50 subjects of age range 17–75 years who were free of cardiovascular disease, hypertension, and diabetes, and who were within the normal range for body mass index. Pulsed wave TDI of LV long-axis motion has been utilised as a technique which allows the measurement of the excursion, peak velocities and timing of systolic and early diastolic motion [31, 33, 42, 43]. TDI was performed at the septal and lateral borders of the mitral annulus and the results of the two walls were analysed separately due to previous evidence that the behaviour of these walls is not identical [44], and also as a check for consistency of the findings.

## Methods

### Subjects

The study design was approved by the Monash Health Human Research and Ethics committee and all clinical investigation was conducted according to the principles expressed in the Declaration of Helsinki. Written informed consent was obtained prospectively from adult subjects recruited as healthy control subjects for other studies. A small number of subjects who had clinically indicated studies were identified retrospectively as fitting the study criteria, and the need to obtain consent from these subjects, or consent from the parent or guardian of any of these subjects who were minors, was waived. Height and weight were measured immediately prior to the echocardiographic study and body surface area (BSA) and body mass index (BMI) were calculated using standard formulae. Blood pressure (BP) was measured during the echocardiographic study with the patient in a supine position. The study group comprised 50 healthy adult subjects between 17 and 75 years. Subjects were eligible if they had no history of cardiac disease, diabetes or hypertension, were on no cardiac medication, had a BMI < 25 kg/m^2^ and had a systolic BP ≤160 and a diastolic BP ≤90 mm Hg at the time of the study. All subjects had a normal LVEF (≥50%), no regional wall motion abnormality and no more than mild valvular disease as assessed by echocardiography.

### Echocardiography

Echocardiography was performed on a Vivid 7 machine (GE Healthcare, Chicago, IL, USA), studies were stored digitally and were measured offline using Xcelera V1.2 L4 SP2 (Philips, Amsterdam, The Netherlands). Apical four- and two-chamber 2-dimensional loops of left ventricular contraction were recorded and used for measurement of LV end-diastolic volume (LVEDV), end-systolic volume and the calculation of the ejection fraction (LVEF) using the biplane method of discs. The length of the LV at end-diastole (LVEDL) from the plane of the mitral annulus to the apical endocardium in the 4- and 2-chamber views was recorded during the measurement of the LVEDV, and the longest dimension from these 2 views has been used [45]. Pulsed-wave TDI was performed in the apical 4-chamber view and TDI signals of longitudinal mitral annular motion were recorded during non-forced end-expiration apnoea at both septal and lateral borders of the mitral annulus after optimising parallel alignment of the ultrasound beam and positioning of the sample volume [44].

**Fig 1:**
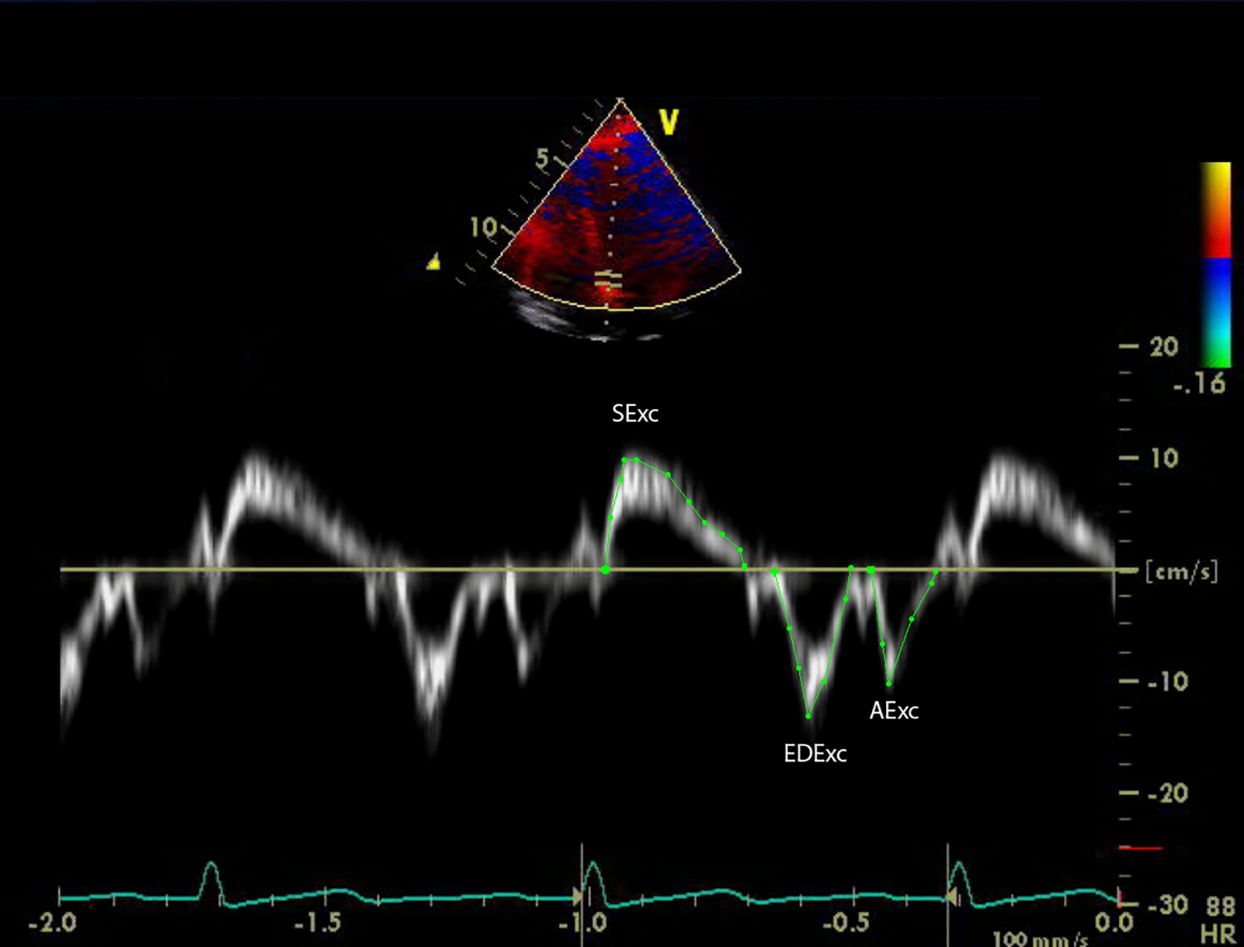
Example of the method for tracing the velocity time integrals of the systolic, early diastolic and atrial contraction tissue Doppler signals from the mitral annulus.

Measurements were made off-line of both systolic and diastolic time intervals from the onset of the QRS complex to the onset and end of the TDI systolic signal (SS) and to the onset, peak and end of the early diastolic signal (EDS). The time interval between the end of the SS and the commencement of the EDS was calculated as a TDI long-axis equivalent of the isovolumic relaxation time (IVRT) [42]. Also calculated from the time intervals were the systolic duration (SDur), the early diastolic duration (EDDur), and the duration of acceleration (EDAT) and the duration of deceleration (EDDT) of the EDS. The heart rate was calculated from the R-R intervals of the relevant TDI signals. Measurements were made from the SS of s`and the velocity time integral (SExc), from the EDS of e' and the velocity time integral (EDExc) and from the atrial contraction signal of the velocity time integral (AExc) as previously described (Fig 1) [25, 33]. The percentage of total diastolic excursion due to early diastolic motion was calculated as EDExc divided by the sum of EDExc and AExc and multiplied by 100.

### Statistical analysis

Statistical analysis was performed using Systat V13 (Systat Software, Chicago, IL, USA). Continuous variables are presented as mean ± SD. A paired t test was used to compare septal and lateral TDI variables within the same individuals. For univariate linear regression analysis the r value is provided and for multivariate analyses, the partial standard correlation coefficient (β) value is provided. The coefficient of determination has been adjusted for the number of terms in multivariate models (adjusted r^2^) and used to estimate the percentage of the variance of a dependent variable explained by that model. The decisions regarding inclusion of variables in multivariate models were based on the questions being addressed. A p value of <0.05 was considered significant.

Univariate linear regression analysis was performed to assess the relationships of age with TDI peak velocities and excursions. Multivariate linear regression analysis was performed to assess the relationships of age with TDI time intervals with adjustment for sex and heart rate. Sex was included in the models of time intervals as a dummy variable (1 for male, -1 for female) because of previous evidence that male sex is accompanied by shorter systolic time intervals [46], and heart rate was included because a higher heart rate is accompanied by shorter systolic time intervals [43, 46] and a shorter IVRT [11, 43].

The contributions of LVEDL to the variances in SExc, EDExc and e`were investigated by univariate linear regression analysis, and the dependence of these relationships on age were examined in multivariate models where age was added to LVEDL and then LVEDL was added to age. SExc was included as the initial independent variable in multivariate models of EDExc on the basis that there is a fundamental, although not necessarily linear, relationship between SExc and EDExc, i.e. the extent of relaxation is dependent on and cannot exceed the extent of the preceding contraction. IVRT has been used in multivariate analyses as a non-invasive measure of the speed of relaxation, this based on previous evidence that IVRT is correlated with the time constant of relaxation in the absence of an increase in left atrial pressure [41], and that left atrial pressure is not expected to be elevated in a cohort of healthy adult subjects [16, 47, 48]. The effect of the speed of relaxation on EDExc independent of the extent of systolic excursion was assessed by the addition of IVRT to models which included SExc. Whether there was an effect of the speed of relaxation on e`independent of the extent of relaxation was tested by the addition of IVRT to the models of e`which included EDExc. The possibility of a contribution of heart rate to the model was also considered in all models containing IVRT. Age was not added until the final step in models of EDExc and e`because of the expected multicollinearity of age with most of the other independent variables. However, models where other independent variables were added after age were also used to determine whether there were age-independent effects of these variables.

## Results

The demographics, ejection fraction and LVEDL of the study group are shown in Table 1. The TDI integrals, e`and EDExc% for the septal and lateral walls are shown in Table 2. The magnitudes of all but one of these variables were larger for the lateral wall, with only AExc being larger for the septal wall.

**Table 1:** Demographic variables in the study group.

Male: Female	22:28
Age (years)	43±18
Height (cm)	166±25
Weight (kg)	63±8
Body surface area (m^2^)	1.73±0.15
Body mass index (kg/m^2^)	21.9±1.8
Blood pressure (mmHg)	118±14/69±11
Ejection fraction (%)	60±4
LVEDL (cm)	9.0±0.8

**Table 2:** Comparison of left ventricular long-axis excursions and velocities for the septal and lateral walls.

	Septal	Lateral	P
SExc (cm)	1.46±0.27	1.56±0.26	0.004
EDExc (cm)	0.97±0.25	1.08±0.29	<0.001
AExc (cm)	0.54±0.13	0.46±0.13	<0.001
e`(cm/s)	10.9±3.4	13.9±4.3	<0.001
EDExc% (%)	63.3±10.5	69.6±9.9	<0.001

Correlations of age with TDI velocities and excursions are shown in Table 3. Similar age correlations were seen for the septal and lateral walls. Age was positively correlated with AExc and inversely correlated with SExc, EDExc, e`and EDExc%. All these correlations remained significant and similar after adjustment for heart rate and sex. Age was positively correlated with diastolic DP (r = 0.43, p = 0.003), septal IVRT (r = 0.56, p<0.001) and lateral IVRT (r = 0.39, p = 0.005), but there was no correlation of diastolic BP with either septal or lateral IVRT, with or without adjustment for heart rate and sex.

**Table 3:** Univariate correlations of age with left ventricular long-axis excursions and e`.

	r	*P*
**Septal**		
SExc	-0.61	<0.001
EDExc	-0.75	<0.001
e`	-0.86	<0.001
AExc	0.65	<0.001
EDExc%	-0.80	<0.001
**Lateral**		
SExc	-0.61	<0.001
EDExc	-0.75	<0.001
e`	-0.87	<0.001
AExc	0.57	<0.001
Lateral EDExc%	-0.79	<0.001

### Long-axis time intervals for the septal and lateral walls

Measured and calculated TDI time intervals for the septal and lateral walls are shown in Table 4. The onset and end of the SS were earlier for the septal than the lateral wall, but SDur was not different between the walls. There were no differences in the times to the onset or peak of the EDS for the two walls but the IVRT, the time to end of the EDS, and the EDDur were all longer for the septal wall. The EDAT was similar for the two walls but there was a longer EDDT for the septal wall, which accounted for the longer EDDur for that wall.

**Table 4:** Comparison of left ventricular long-axis time intervals for the septal and lateral walls.

	Septal	Lateral	P
Heart rate	63±10	63±10	NS
Q—onset SS (ms)	77±19	83±18	0.019
Q–end septal SS (ms)	374±27	382±26	<0.001
SDur (ms)	297±22	296±30	NS
Q–onset EDS (ms)	456±36	454±35	NS
Q–peak EDS (ms)	513±36	514±36	NS
Q–end EDS (ms)	624±47	604±44	<0.001
IVRT (ms)	82±26	72±19	0.001
EDDur (ms)	165±30	150±29	<0.001
EDAT (ms)	58±14	61±17	NS
EDDT (ms)	111±27	90±28	<0.001

### Age and long-axis time intervals

In multivariate models including sex and heart rate, male sex was associated with shorter time intervals of septal Q to the onset, peak and end of the EDS, but none of the other time intervals, and heart rate was inversely correlated with all the septal time intervals and for all the lateral time intervals except Q to the onset of the EDS and EDDur. The standard partial correlation coefficients of age with the time intervals, after adjustment for sex and heart rate, are shown in Table 5. There was no correlation of age with any of the systolic time intervals, but age was positively correlated with the time from the Q wave to the onset and the end of the EDS and with the IVRT for both walls. There was no correlation of age with septal EDDur or its components of EDAT and EDDT, but there was a complex relationship of age with the lateral EDS time intervals; lateral EDDur was longer, EDAT was shorter and EDDT was longer with increasing age.

**Table 5:** Partial standard correlation coefficients of age with time intervals after adjustment for sex and heart rate.

	Β	*P*
**Septal**		
Q–onset SS	0.23	0.10
Q–end SS	-0.03	0.83
SDur	-0.23	0.08
Q–onset EDS	0.41	<0.001
Q–peak EDS	0.38	0.001
Q–end EDS	0.39	0.001
IVRT	0.61	<0.001
EDDur	0.16	0.29
EDAT	-0.07	0.67
EDDT	0.17	0.27
**Lateral**		
Q–onset SS	0.20	0.17
Q–end SS	0.03	0.75
SDur	0.01	0.93
Q–onset EDS	0.31	0.003
Q–peak EDS	0.12	0.23
Q–end EDS	0.49	<0.001
IVRT	0.52	<0.001
EDDur	0.36	0.019
EDAT	-0.40	0.006
EDDT	0.61	<0.001

### Relations of LVEDL with age, SExc, EDExc and e`

On univariate analysis, LVEDL was inversely correlated with age (r = -0.61, p<0.001) and BSA (r = 0.62, p<0.001) and was larger in males (p = 0.003). On multivariate analysis, LVEDL was positively correlated with BSA (β = 0.50, p<0.001) and inversely correlated with age (β = -0.49, p<0.001), the effect of sex was no longer significant, and together BSA and age explained 59% of the variance in LVEDL.

LVEDL was positively correlated with septal and lateral SExc (r = 0.46 & r = 0.70), septal and lateral EDExc (r = 0.51 & r = 0.66) and septal and lateral e`(r = 0.54 & r = 0.66, p<0.001 for all LVEDL correlations). In multivariate models in which age was added to LVEDL, the addition of age substantially improved the variances explained for septal and lateral SExc, EDExc and e`. In combination with age, LVEDL became a minor contributor in the models of lateral SExc and EDExc and e`and was no longer a significant contributor to the models of septal SExc, EDExc and e`. When age was added to models of e`which included LVEDL, there were improvements of 34–46% in the variance of e`explained. On the other hand, the addition of LVEDL to age increased the variance of lateral e`explained by only 7%, indicating that there was only a small age-independent component of the contribution of LVEDL to the prediction of lateral e`.

### EDExc as a dependent variable

**Fig 2:**
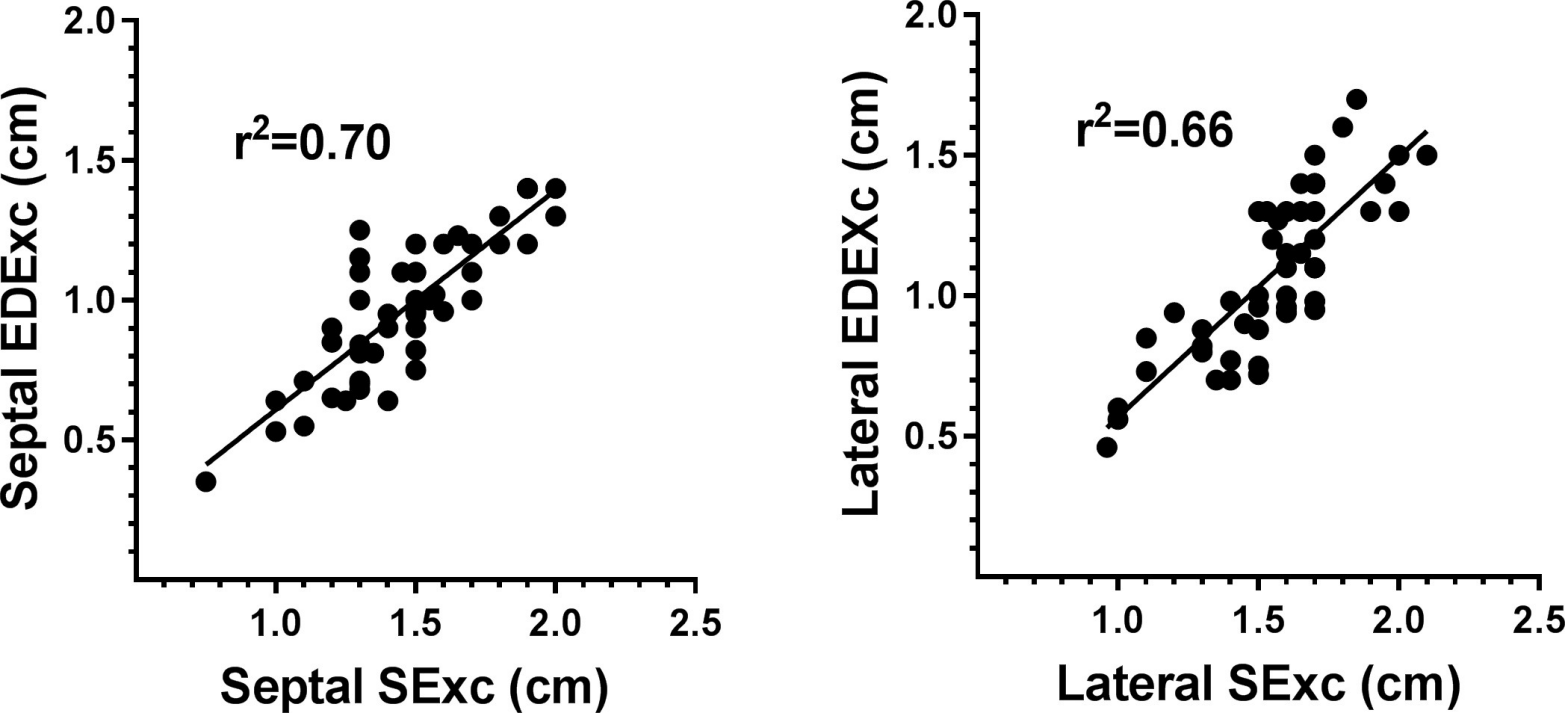
Scatter plots showing the relationships of EDExc with SExc for the septal and lateral walls.

Selected univariate correlates of septal and lateral EDExc are shown in Table 6 and scatter plots showing the relationship of EDExc with SExc are presented in Fig 2. Septal and lateral EDExc were both positively correlated with BSA, SExc and LVEDL, and inversely correlated with age, IVRT and heart rate. The scatter plots of EDExc versus SExc are shown in Table 2. Lateral EDExc was also inversely correlated with diastolic BP, whereas an inverse correlation of septal EDExc with diastolic BP was weaker and only borderline significant. In multivariate models none of sex, BSA or LVEDL were independent predictors of septal or lateral EDExc when combined with the respective SExc and these variables were not included in further modelling. In combination, IVRT and heart rate were independent determinants of EDExc (p<0.01 for both variables and both walls) and explained 43–67% of the variances of EDExc.

**Table 6:** Univariate correlations of septal and lateral EDExc.

	Septal EDExc		Lateral EDExc	
	r	P	R	P
BSA	0.41	0.003	0.43	0.002
SExc	0.84	<0.001	0.82	<0.001
Diastolic BP	-0.28	0.05	-0.40	0.004
IVRT	-0.73	<0.001	-0.43	0.002
Heart rate	-0.37	0.008	-0.37	0.008
Age	-0.75	<0.001	-0.77	<0.001
LVEDL	0.51	<0.001	0.66	<0.001

Multivariate models of septal and lateral EDExc constructed with the sequential addition of SExc, diastolic BP, IVRT and heart rate are shown in Table 7. Models including age and the significant independent predictors from the above variables are also shown in Table 7. For both the septal and lateral walls, the major portion of EDExc was explained by the respective SExc (66–70% of the variances). Diastolic BP was not a correlate of septal EDExc when included with SExc and is not shown in the multivariate model of septal EDExc. Diastolic BP was an independent correlate of lateral EDExc when added to SExc, but only made a small contribution (3%). When IVRT and heart rate were both added to SExc, there was only an increase of 8% in the variance of septal EDExc explained. When IVRT and heart rate were both added to SExc and diastolic BP there was only a 4% increase in the variance of lateral EDExc explained. In subsequent analysis, that there was only a minor contribution of IVRT to SExc in the prediction of EDExc could be explained by the presence of univariate correlations of SExc with IVRT for both the septal and lateral walls (r = 0.71 & r = 0.35, respectively, p<0.02 for both).

**Table 7:** Multivariate models of septal and lateral EDExc.

	Independent variable	Univariate r	Multivariate β	P value in multivariate model	Cumulative adjusted r^2^
**Septal EDExc**	Septal SExc	0.84	0.52	<0.001	0.70
	IVRT`	-0.73	-0.36	0.001	0.73
	Heart rate	-0.37	-0.26	0.001	0.78
**Septal EDExc**	Septal SExc	0.84	0.44	<0.001	0.70
	IVRT`	-0.73	-0.27	0.007	0.73
	Heart rate	-0.37	-0.19	0.011	0.78
	Age	-0.75	-0.26	0.003	0.82
**Lateral EDExc**	Lateral SExc	0.82	0.64	<0.001	0.66
	Diastolic BP	-0.40	-0.16	0.033	0.69
	IVRT`	-0.43	-0.27	0.002	0.70
	Heart rate	-0.37	-0.26	0.003	0.75
**Lateral EDExc**	Lateral SExc	0.82	0.49	<0.001	0.66
	Diastolic BP	-0.40	-0.09	0.23	0.69
	IVRT`	-0.43	-0.18	0.036	0.70
	Heart rate	-0.37	-0.18	0.028	0.75
	Age	-0.77	-0.31	0.002	0.80

After age was added to the model of septal EDExc which included SExc, IVRT and heart rate, all the independent variables continued to make significant contributions and there was only a further increase of 4% in the variance of EDExc explained. After adding age to the model of lateral EDExc including SExc, diastolic BP, IVRT and heart rate, all the independent variables except for diastolic BP still made significant contributions and there was only a further increase of 5% in the variance of EDExc explained. The partial correlation coefficients of SExc, IVRT, heart rate and age all decreased after the addition of age, with SExc remaining the largest contributor in the models of both septal and lateral EDExc. As the sole independent variable, age accounted for 55% of the variance in septal EDExc and 59% of the variance in lateral EDExc. When SExc was added to age there were increases of 19–23% in the variances of EDExc explained compared to age as the sole independent variable, demonstrating that there were substantial age-independent contributions of SExc to the prediction of EDExc for both LV walls.

### e`as a dependent variable

**Fig 3:**
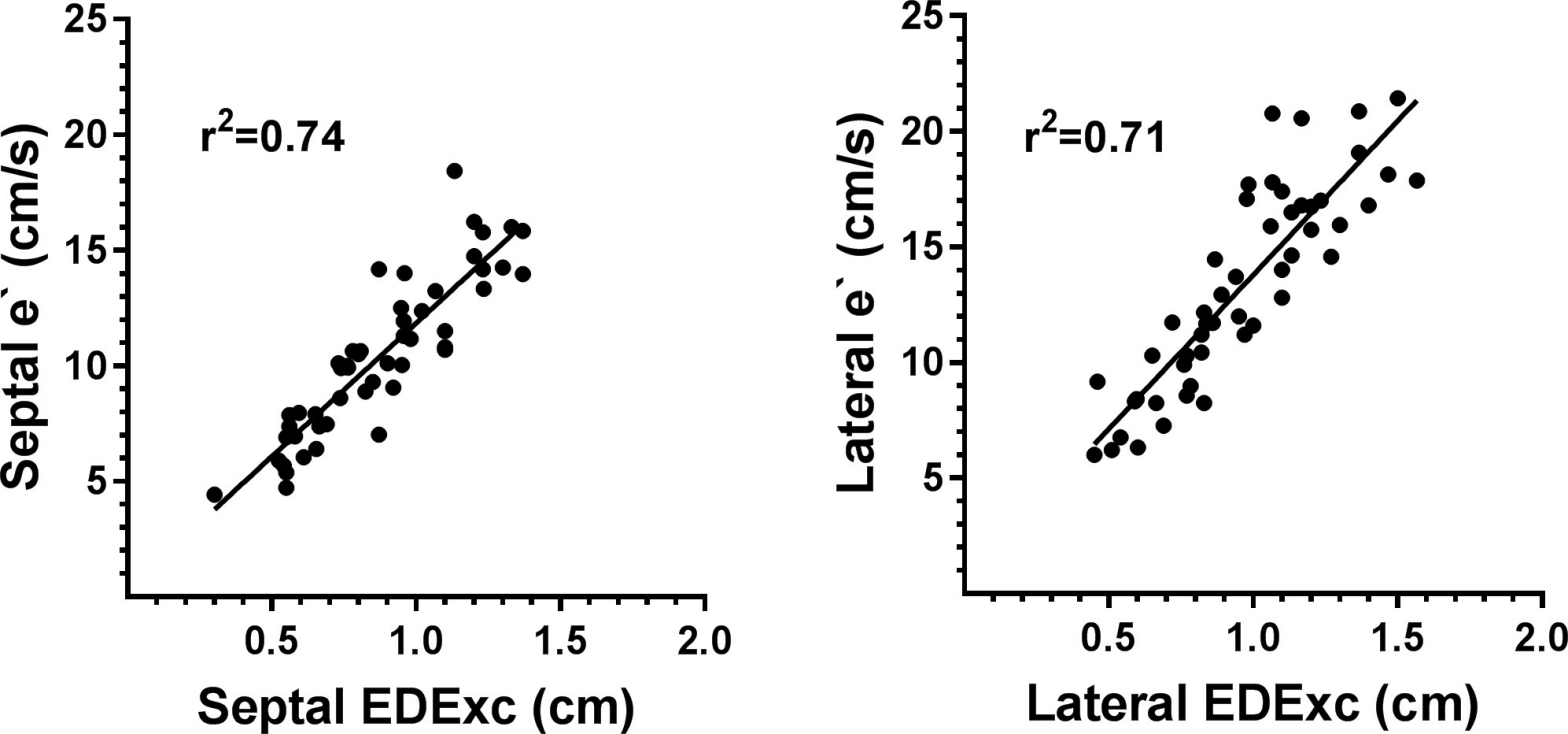
Scatter plots showing the relationships of e`with EDExc for the septal and lateral walls.

Univariate correlations of e`with selected variables are shown in Table 8 and scatter plots showing the relationship of e`with EDExc are presented in Fig 3. Both septal and lateral e`were positively correlated with BSA, SExc, EDExc and LVEDL and inversely correlated with diastolic BP, IVRT and age. Scatter plots of the relationship between e`and EDExc for the septal and lateral walls are shown in Fig 2. Lateral e`was also inversely correlated with heart rate, but an inverse correlation of septal e`with heart rate was only borderline significant. In multivariate models neither BSA nor LVEDL were independent predictors of septal or lateral e`once the respective EDExc was included in the model and these variables were not included in subsequent modelling.

**Table 8:** Univariate correlations of septal and lateral e`.

	Septal e`		Lateral e`	
	R	P	r	p
Body surface area	0.30	0.034	0.38	0.007
SExc	0.78	<0.001	0.69	<0.001
EDExc	0.86	<0.001	0.84	<0.001
Diastolic BP	-0.43	0.002	-0.40	0.004
IVRT	-0.75	<0.001	-0.37	0.007
Heart rate	-0.26	0.064	-0.39	0.005
Age	-0.86	<0.001	-0.87	<0.001
LVEDL	0.54	<0.001	0.67	<0.001

Multivariate models of septal and lateral e`which demonstrate the effects of sequential addition of EDExc, diastolic BP, IVRT and heart rate are shown in Table 9. For both the septal and lateral walls, the major portions of the variances in e`were explained by the respective EDExc (71–74%). The addition of diastolic BP to SExc resulted in an increase in 3% of the variance of septal e`explained, but BP was not a significant contributor in the model of lateral e`. A further 3% of the variance of septal e`was explained by the addition of IVRT to EDExc and diastolic BP, but IVRT was not a contributor to the prediction of lateral e`. Although both IVRT and heart rate in combination were significant contributors to both septal and lateral e`in the absence of other independent variables (p<0.05 for all), heart rate made no contribution to models of e`which also included EDExc as an independent variable. The lack of contribution of IVRT when added to EDExc in the prediction of e`could be explained by the presence of inverse correlations of EDExc with IVRT on univariate analysis for both the septal and lateral walls (see Table 6).

**Table 9:** Multivariate models of septal and lateral e`.

	Independent variable	Univariate r	Multivariate β	P value in multivariate model	Cumulative adjusted r^2^
**Septal e`**	Septal EDExc	0.86	0.63	<0.001	0.74
	Diastolic BP	-0.43	-0.19	0.006	0.77
	IVRT`	-0.75	-0.25	0.009	0.80
**Septal e`**	Septal EDExc	0.74	0.33	0.001	0.74
	Diastolic BP	-0.43	-0.09	0.10	0.77
	IVRT`	-0.25	-0.25	0.001	0.80
	Age	-0.86	-0.45	<0.001	0.88
**Lateral e`**	Lateral EDExc	0.84	0.81	<0.001	0.71
	Diastolic BP	-0.40	-0.08	0.37	0.71
	IVRT`	-0.37	-0.02	0.85	0.70
**Lateral e`**	Lateral EDExc	0.84	0.43	<0.001	0.71
	Age	-0.87	-0.54	<0.001	0.82

Including age with EDExc, diastolic BP and IVRT explained a further 8% of the variance of septal e`and including age with EDExc explained a further 11% of the variance in lateral e`. There were concomitant reductions in the partial correlation coefficients of both variables and age was the major contributor in these models. When EDExc was added to age in models of e`there was an increase of 7–11% in the variances of e`explained compared to age as the sole independent variable, indicating the presence of an age-independent contribution of EDExc to the prediction of e`.

## Discussion

A linear decrease in e`in adults during aging has now been a consistent finding in many studies, but the mechanisms underlying the age-related decrease in e`have remained uncertain. A causal relationship between age-related slowing of relaxation and the decrease in e`had been assumed by some investigators [12, 16], however, the possibility of involvement of alternative or additional mechanisms in the determination of e`has also been considered [24, 27, 33]. In the present study, the mechanisms underlying the aging effects on e`have been investigated in a cohort of healthy adult subjects, and there are a number of new findings which applied to both the septal and lateral walls. First, in the setting of a linear decrease in e`from the 3rd to 8th decade similar to that seen in previous studies, there was also a linear decrease in EDExc with age, and there was a close relationship between e`and EDExc. Second, SExc also decreased with age, a finding consistent with previous findings using M-mode and CMR techniques, but which has not been previously considered as a determinant of aging-related decreases in EDExc and e`. Third, there was a decrease of LVEDL with age and LVEDL was positively correlated with SExc, EDExc and e`, supporting the possibility of structural LV remodelling having a direct relationship with both long-axis contraction and early diastolic motion. Fourth, SExc was the major determinant of EDExc, via a combination of age-dependent and age-independent mechanisms. Fifth, EDExc was a major determinant of e`, also via age-dependent and age-independent mechanisms. Sixth, while the expected positive correlation of IVRT with age was observed, implying an aging-related slowing of LV relaxation, IVRT was also inversely correlated with SExc, was only a minor determinant of EDExc after taking SExc into account, and was an inconsistent determinant of e`after taking EDExc into account. The above findings demonstrate previously under-appreciated relationships between e`and the extent of long-axis contraction and relaxation, with aging-related effects on long-axis function partly explained by structural long-axis LV remodelling. Furthermore, not only was a decrease in the speed of relaxation not the major factor in the aging-related reductions of EDExc and e`, but it played only a minor role after adjusting for SExc and EDExc, respectively.

In the current study a novel approach was utilised to investigate aging-related reductions in LV early diastolic long-axis motion. First, if two diastolic variables were both correlated with age there has been no assumption that this necessarily means the mechanisms underlying the aging-related changes in the two variables are the same. Second, given that the extent of contraction is both an essential and limiting factor to any subsequent excursion during diastole, SExc was the first independent variable entered in multivariate modelling investigating the possible determinants of EDExc. Third, EDExc was considered as an essential element and thus likely determinant of e`, and was therefore the first independent variable entered in multivariate modelling investigating the possible determinants of e`. Fourth, the percentage contributions of the independent variables to the prediction of EDExc and e`were determined using stepwise addition of the independent variables, with ordering based on the hypotheses being tested rather than the closeness of any univariate correlations. Fifth, age was not added until the final step in most of the multivariate analyses to avoid confounding of the analysis by multicollinearity of age with the other independent variables. Sixth, multivariate modelling in which independent variables were added to age was also performed to determine if there were age-independent effects of these variables.

### Relationships of SExc and EDExc with age

A key finding of this study is that age was inversely correlated with both septal and lateral SExc, indicating an age-related progressive reduction in the extent of LV long-axis contraction in our subject group. Consistent findings regarding long-axis contraction in subjects of a similar age range have been previously reported in studies in which mitral annular plane systolic excursion (averaging measurements from multiple annular sites using either M-mode or cardiac magnetic resonance images) was used to assess long-axis contraction [30, 32, 37–40]. There was also an aging-related reduction in EDExc in the present study, this being consistent with previous studies using either M-mode [32] or TDI [33] techniques. That EDExc is contingent on there being a preceding contraction, and cannot be more than SExc, was the basis on which SExc was the first variable considered in this study when investigating the determinants of EDExc. A linear correlation between SExc and EDExc could not be assumed given that AExc also increases with age, but was manifest for both LV walls, with 66–70% of the variances in septal and lateral EDExc explained by SExc alone. Therefore, SExc was the major determinant of the variability in EDExc in this healthy cohort and a causal relationship for this finding is likely. That SExc must be equal or nearly equal to the sum of EDExc and AExc for any cardiac cycle, and that both SExc and EDExc decrease with age, indicates that the observed increase in AExc with aging in the present study was of lower magnitude than the decrease in EDExc.

### Relation of LVEDL to age and LV long-axis TDI variables

LVEDL was inversely correlated with age in the present study, a finding which is consistent with previous cross-sectional data that LVEDL decreases during aging [32, 34–36]. While loading conditions need to be considered as a possible contribution to this aging-related change in LVEDL, there is no reason to postulate a reduction in preload via a decrease in intravascular volume or LV filling with age in this healthy non-fasting cohort. Indeed, available evidence from invasive studies suggests no change in left atrial pressure [16, 47, 48], and an increase in LV end-diastolic pressure [49, 50] during the aging process. An additional finding from previous cross-sectional studies was that the reductions in LVEDL with aging were not accompanied by changes in LV end-diastolic short-axis dimension [32, 34, 36], and so in conjunction with the evidence that preload reduction is unlikely in our cohort, the reduction in LVEDL with age appears to reflect a relatively specific long-axis remodelling process.

LVEDL was positively correlated with SExc, EDExc and e`for both LV walls in the present study. This reflects only partial agreement with a previous report in which positive univariate correlations of LVEDL with e`and EDExc were significant for the lateral wall but not for the septal wall [33], but at least two differences between the characteristics of the subject groups in the studies could have contributed to the divergence in findings. First, the present study group included a wider age range than the previous study, which only included adults < 55 years, and this age limitation may have reduced the power of the previous study to show a relationship between LVEDL and septal e`, both of which continue to decrease after the age of 55 years. A second difference was that subjects with a BMI of >25 kg/m^2^ were excluded in the present study, whereas in the previous study subjects with BMI values of up to 30 kg/m^2^ were included. This may be important as the lack of a significant univariate correlation of LVEDL with septal e`on univariate analysis in the previous study appeared to be due to confounding by the inverse correlation of septal e`with BMI, in conjunction with a positive correlation of LVEDL with BMI.

The relationships of LVEDL with SExc, EDExc or e`can be mostly attributed to the age-related change in LVEDL in the present study. Thus, after addition of age to LVEDL in models of septal SExc, EDExc or e`, LVEDL was no longer a significant contributor to the prediction of the dependent variables, whereas after the addition of age to LVEDL for the lateral wall variables, LVEDL became only a minor contributor in the models of lateral EDExc and e`. Nevertheless, these findings are consistent with aging-related structural remodelling being one of the mechanisms by which aging effects on long-axis function may be explained. The aging-related reductions in SExc were only partly explained by structural LV remodelling and therefore aging must also be associated with reductions in SExc by a non-remodelling related reduction of contraction. An explanation at the ultrastructural level for these observations is not currently possible as information regarding the effects of healthy aging on LV interstitial tissue, myocytes and myocyte function in adult humans is limited.

### IVRT as a measure of speed of relaxation

IVRT is the time interval between aortic valve closure and mitral valve opening and its magnitude is dependent on the aortic closing pressure, the rate of pressure fall in the left ventricle after aortic valve closing, and the left atrial pressure [51]. In the present study which was performed in subjects without hypertension, it has been assumed that any aortic pressure effects on the IVRT were also related to age, that left atrial pressure would not vary systematically with age [16, 47, 48], and therefore that IVRT could be used as a guide to the speed of LV pressure fall. A number of different non-invasive Doppler methods using the onset of transmitral flow have been previously used to measure IVRT [51], but in the present study IVRT was measured using pulsed wave TDI from the end of the systolic signal to the beginning of the early diastolic signal, a method which has been previously described [42]. While it is possible that the absolute magnitude of IVRT may vary depending on the specific method of measurement, the magnitude of the positive correlation of IVRT with age in the present study is consistent with a previous study in subjects of comparable age range which utilised a more standard technique [11].

### Relationship of speed of relaxation with EDExc

IVRT was added to models of EDExc which included SExc to test for a role of slowing of relaxation in the decrease in EDExc with aging which was independent of any concomitant reduction in contraction. Heart rate was also added in these models given that heart rate is an inverse correlate of IVRT [11, 33, 52], in conjunction with evidence from the current study that it was not only inversely correlated with IVRT, but that it also contributed to the prediction of EDExc. However, while IVRT and heart rate together were both significant inverse correlates of EDExc, they made only small additional contributions to the prediction of the variances in septal and lateral EDExc when added to SExc. Thus, a total of 73–78% of the variances in EDExc could be explained by the combination of SExc, IVRT and heart rate, compared to the 66–70% explained by SExc alone. That the presence of IVRT correlations with age and EDExc did not have to indicate a substantial causal relationship between slowing of relaxation during aging and the reduction in EDExc was suggested by the coexistence of a correlation between IVRT and SExc, because a decrease in speed of relaxation is not an explanation for a decrease in SExc. These results indicate that after considering systolic excursion, the decrease in the speed of relaxation was no more than a minor contributor to the reduction of EDExc during aging in this study cohort.

### Age-dependent and independent contributions to EDExc

Age as a single independent variable explained 55–59% of the variances in EDExc and therefore did not account for all the contributions from SExc, IVRT and heart rate. After the addition of age as the final step in the models of EDExc including SExc, IVRT and heart rate, there was a further increase in the variance of EDExc explained. While there were reductions in the partial standard correlation coefficients of all 4 variables, each of the 4 variables still made independent contributions to the prediction of EDExc. Therefore SExc and IVRT both appear to contribute to a reduction in EDExc by age-related and age-unrelated mechanisms. SExc was not only the best individual predictor of EDExc as a single variable but it remained the best individual predictor of EDExc after the addition of IVRT, heart rate and age (Table 6). The major contribution of SExc to the prediction of EDExc was by an age-related mechanism, but 18–23% of the variances in EDExc were explained by SExc via an age-independent mechanism, this likely related in part to variation in SExc also being a reflection of individual variations in the extent of LV long-axis contraction.

### Relationship of e`with EDExc and age

In the current study age was inversely correlated with e`for both the septal and lateral walls, an observation consistent with the findings of many previous studies [8–10, 12–15, 53]. That the reductions of e`and EDExc with aging were closely related was demonstrated by the combination of findings that there were inverse correlations of age with EDExc and that there were positive correlations of e`with EDExc for both walls. An inverse correlation of age with EDExc [32, 33] and a positive correlation of e`with EDExc [31, 33] have been previously described, but the relationship of e`with EDExc has been largely ignored in TDI studies over the last 20 years. Age and EDExc were both correlated with e`on univariate analysis and combination of these two independent variables in models of e`demonstrated that age made small EDExc-independent contributions and EDExc made small age-independent contributions to the prediction of e`in these models.

### Relationship of speed of relaxation with e`

Although it is believed by some investigators that the reduction in e`with age is caused by an age-related reduction in the speed of LV relaxation, operating at least in part at the level of the myocyte [12, 16], increasing evidence suggests that e`is determined by more than just the LV relaxation rate [15, 24–27, 33]. Indeed, the closest correlation of e`in the present study was with the extent of early diastolic long-axis motion. For this reason EDExc was the first independent variable entered in models of e`and the additional contributions of IVRT and heart rate to models of e`containing EDExc were investigated to see if speed of relaxation accounted for some of the variability in e`independent of EDExc. However, in these models IVRT was only a minor contributor to the prediction of septal e`and was not a significant contributor to the prediction of lateral e`. These results indicate that, after accounting for early diastolic excursion, which in turn is predominantly determined by systolic excursion, the aging-related decrease in the speed of relaxation is no more than a minor contributor to the reduction of e`which accompanies aging.

### Age effects on the timing of long-axis function

An important element of the present study was investigation of the effects of aging on the timing of LV long-axis cardiac motion. There is evidence from a number of animal studies that aging results in prolongation of the duration of myocardial contraction [54, 55], and this prolongation could also have relevance to the timing of diastolic events [51]. However, there is divergent evidence from human studies regarding whether aging is associated with prolongation of the left ventricular ejection [1, 56–58] and the Q wave to aortic valve closure times [11]. The lack of any substantial aging effects on long-axis contraction duration in the present study could reflect in part that our group did not include very elderly subjects and that subjects with hypertension were excluded. The apparent discrepancy compared to myocardial bench-top studies could also reflect differences in the behaviour of isolated myocardium versus the behaviour of the heart in the intact circulation. It is also important to consider that contraction is not simultaneous in myocardial fibres in the left ventricle and is not even simultaneous for longitudinal fibres in a single wall [59, 60], in which case assessment at the mitral annulus of the duration of LV long-axis contraction could fail to detect an aging-related prolongation of contraction in individual myocytes or myocardial fibres.

There is evidence from myocardial studies in animals that aging is associated with prolongation of relaxation times [54, 61], and human evidence that aging is accompanied by prolongation of the IVRT [3, 11, 62]. Consistent with these reports, there was not only an aging-related prolongation of IVRT, but there was a delay in the times to the onset and end of the EDS for both walls in the present study. This therefore represents a systematic delay in the whole process of early diastolic long-axis motion. On the other hand, there was no change in septal EDDur, EDAT or EDDT with age in our study group, whereas there was a complex relationship of age with lateral wall times, with age positively correlated with EDDur and EDDT, but inversely correlated with EDAT. It can therefore be concluded that prolongation of IVRT is the only consistent contributor to the aging-related delay to the end of early diastolic motion. Furthermore, reductions in e`with age are not dependent on changes in EDAT or EDDur. From a mechanistic perspective, it cannot be assumed that aging-related prolongation of IVRT is solely due to slowing of myocyte relaxation given that incoordination of contraction is a common cause of a prolonged IVRT in disease processes [51]. Furthermore, diastolic LV dyssynchrony with aging has been previously described using nuclear imaging of short-axis function [6] and with pulsed wave TDI assessment of LV long-axis function [63]. An important concept regarding the timing of any TDI measurements at the mitral annulus is that they represent the net effects of all the myocytes along the wall, some of which could be contracting while others are relaxing.

### Limitations

This was a cross-sectional study and therefore has limitations common to all observational studies, particularly with regard to issues of causation. However, longitudinal data which could address the aims of this study would take most of a lifetime to accrue and are thus not feasible. There were no ECG or regional wall motion abnormalities in the study subjects but there was not systematic testing for the presence of coronary disease and so asymptomatic coronary disease could not be excluded. The study group also had the limitation of not being population based. The above limitations could be important if the findings were discrepant with previous studies of aging-related LV effects, however, the relationships of age with e`, SExc, LVEDL and IVRT were consistent with and similar in magnitude to previous reports. Lastly, this was a relatively small study and as with all new findings, confirmation is required.

## Conclusions

The present study investigated the mechanisms underlying the changes in early diastolic motion which occur during aging. The magnitude of SExc, EDExc, e`and LVEDL all decreased during aging between the end of the second and the eighth decade. The major contributor to the prediction of EDExc was SExc and the major component of this was an aging-related decrease in long-axis contraction. In turn, EDExc was the major contributor in the prediction of e`and this was predominantly due to an aging-related reduction of EDExc. Speed of relaxation, as assessed by IVRT, made only a minor contribution to the prediction of EDExc when added to SExc, and was not a consistent determinant of e`when added to EDExc. Thus, despite correlations of IVRT with early diastolic variables, the reductions in both the extent and peak velocity of early diastolic motion appear to be largely independent of the concomitant delay in the onset of motion. Furthermore, there were no consistent aging-related changes in the duration of EDAT, which therefore cannot play a major role in the reduction in e`with aging. The above findings demonstrate that valuable information about LV long-axis relaxation is provided by consideration of the extent of motion during both systole and diastole. A structural remodelling component of the findings regarding age and LV long-axis function can be considered likely given the positive correlations of LVEDL with SExc, EDExc, e`and age.

## Supporting information

S1 File Healthy aging study 2018. (XLS)

## Abbreviations

AExc: atrial contraction mediated excursion of the mitral annulus
BMI: body mass index
BP: blood pressure
BSA: body surface area
e`: peak velocity of early diastolic mitral annular motion
EDAT: duration of acceleration of early diastolic mitral annular motion
EDDT: duration of deceleration of early diastolic mitral annular motion
EDDur: total duration of early diastolic mitral annular motion
EDExc: early diastolic mitral annular excursion
EDS: early diastolic signal
IVRT: isovolumic relaxation time
LV: left ventricular
LVEDL: left ventricular end-diastolic length
SDur: duration of systolic mitral annular motion
SExc: systolic mitral annular excursion
SS: systolic signal
