# Correction: Aging and the relationships between long-axis systolic and early diastolic excursion, isovolumic relaxation time and left ventricular length—Implications for the interpretation of aging effects on e`

**DOI:** 10.1371/journal.pone.0221610

**Published:** 2019-08-20

**Authors:** Roger E. Peverill

In the Results subsection of the Abstract, there is an error in the third sentence. The correct sentence is: IVRT increased with age and on univariate analysis was not only inversely correlated with EDExc and e`, but also with SExc.

In the first paragraph of the Relations of LVEDL with age, SExc, EDExc and e`subsection of the Results, there is an error in the first sentence. The correct sentence is: On univariate analysis, LVEDL was inversely correlated with age (r = -0.61, p<0.001), positively correlated with BSA (r = 0.62, p<0.001), and was larger in males (p = 0.003).

In the first paragraph of the EDExc as a dependent variable subsection of the Results, the third sentence should be removed. The correct first paragraph of the EDExc as a dependent variable subsection of the Results is: Selected univariate correlates of septal and lateral EDExc are shown in Table 6 and scatter plots showing the relationship of EDExc with SExc are presented in Fig 2. Septal and lateral EDExc were both positively correlated with BSA, SExc and LVEDL, and inversely correlated with age, IVRT and heart rate. Lateral EDExc was also inversely correlated with diastolic BP, whereas an inverse correlation of septal EDExc with diastolic BP was weaker and only borderline significant. In multivariate models none of sex, BSA or LVEDL were independent predictors of septal or lateral EDExc when combined with the respective SExc and these variables were not included in further modelling. In combination, IVRT and heart rate were independent determinants of EDExc (p<0.01 for both variables and both walls) and explained 43–67% of the variances of EDExc.

In the second paragraph of the EDExc as a dependent variable subsection of the Results, there is an error in the eighth sentence. The correct sentence is: In subsequent analysis, that there was only a minor contribution of IVRT to SExc in the prediction of EDExc could be explained by the presence of univariate inverse correlations of SExc with IVRT for both the septal and lateral walls (r = -0.71 & r = -0.35, respectively, p<0.02 for both).

In the first paragraph of the e`as a dependent variable subsection of the Results, the third sentence should be removed. The correct first paragraph of the e`as a dependent variable subsection of the Results is: Univariate correlations of e`with selected variables are shown in Table 8 and scatter plots showing the relationship of e`with EDExc are presented in Fig 3. Both septal and lateral e`were positively correlated with BSA, SExc, EDExc and LVEDL and inversely correlated with diastolic BP, IVRT and age. Lateral e`was also inversely correlated with heart rate, but an inverse correlation of septal e`with heart rate was only borderline significant. In multivariate models neither BSA nor LVEDL were independent predictors of septal or lateral e`once the respective EDExc was included in the model and these variables were not included in subsequent modelling.

In the second first paragraph of the e`as a dependent variable subsection of the Results, there is an error in the third sentence. The correct sentence is: The addition of diastolic BP to EDExc resulted in an increase in 3% of the variance of septal e`explained, but BP was not a significant contributor in the model of lateral e`.

In Table 5, the heading of the second column is incorrect. Please see the correct Table 5 here.

**Table 5:** Partial standard correlation coefficients of age with time intervals after adjustment for sex and heart rate.

	β	*p*
**Septal**		
Q–onset SS	0.23	0.10
Q–end SS	-0.03	0.83
SDur	-0.23	0.08
Q–onset EDS	0.41	<0.001
Q–peak EDS	0.38	0.001
Q–end EDS	0.39	0.001
IVRT	0.61	<0.001
EDDur	0.16	0.29
EDAT	-0.07	0.67
EDDT	0.17	0.27
**Lateral**		
Q–onset SS	0.20	0.17
Q–end SS	0.03	0.75
SDur	0.01	0.93
Q–onset EDS	0.31	0.003
Q–peak EDS	0.12	0.23
Q–end EDS	0.49	<0.001
IVRT	0.52	<0.001
EDDur	0.36	0.019
EDAT	-0.40	0.006
